# Ultra-processed food consumption, socio-demographics, and diet quality in Australian adults – CORRIGENDUM

**DOI:** 10.1017/S1368980021004067

**Published:** 2022-01

**Authors:** Laura Marchese, Katherine M. Livingstone, Julie L. Woods, Kate Wingrove, Priscila Machado

A previous version of this article contained some incorrect author data. This has been corrected.
